# High Efficient CO_2_ Separation at High Pressure by Grain-Boundary-Controlled CHA Zeolite Membrane Investigated by Non-Equilibrium Molecular Dynamics

**DOI:** 10.3390/membranes13030278

**Published:** 2023-02-26

**Authors:** Fumiya Hirosawa, Masaya Miyagawa, Hiromitsu Takaba

**Affiliations:** 1 Graduate School of Engineering, Kogakuin University, Tokyo 192-0015, Japan; 2 Department of Environmental Chemistry and Chemical Engineering, School of Advanced Engineering, Kogakuin University, Tokyo 192-0015, Japan

**Keywords:** chabazite zeolite membrane, high pressure CO_2_/CH_4_ separation, molecular dynamics

## Abstract

The CO_2_ permeability and selectivity of CHA-type zeolite membranes in the separation of a CO_2_/CH_4_ mixture gas at high pressure were evaluated using non-equilibrium molecular dynamics (NEMD). It was found that in a perfectly crystalline, defect-free CHA membrane, the adsorption of CH_4_, which diffuses slowly in the pores, hinders CO_2_ permeation. Therefore, an increase in the amount of CH_4_ adsorbed at high pressure decreases the CO_2_ permeability and significantly reduces the CO_2_ selectivity of the CHA membrane. CHA membranes with grain boundaries parallel to the permeation direction were found to show higher CO_2_ selectivity than perfectly crystalline CHA membranes at high pressure, as the blocking effect of CH_4_ on CO_2_ permeation occurring within the grain boundary is not significant. This paper is the first to show that the CO_2_ permeability of CHA membranes with controlled grain boundaries can exceed the intrinsic performance of fully crystalline zeolite membranes at high pressure.

## 1. Introduction

Greenhouse gases are considered to be the main cause of global warming and the reduction of CO_2_ emissions into the atmosphere is necessary; CCS (carbon dioxide capture and storage) and CCUS (carbon dioxide capture, utilization, and storage) are attracting worldwide attention, and CO_2_ separation is a fundamental technology for them. Natural gas refining is a well-known CO_2_ separation process [1]. Associated gas produced from gas fields contains CO_2_, so a process to purify it from methane is essential for the utilization of natural gas. The gas concentration of the associated gas varies from producing area to producing area, and the CO_2_ concentration is known to vary greatly from 10 to 90% [2].

Chemical absorption is the current process in natural gas plants but is expensive for the regeneration of the absorbent when separating high concentrations of CO_2_. The membrane separation is a process driven by the chemical potential gradient (concentration and pressure). Membrane separations are simple devices and can be operated continuously so that the pressure of gas emanating from gas fields can be used as the driving force, such as in natural gas plants, which are the target of separation. Polymer membranes have already been commercialized in the field of natural gas, but there are issues with CO_2_ separation performance and membrane durability [3]. Furthermore, they show a high affinity for aromatic hydrocarbons, which are present in trace amounts in natural gas, making their practical application difficult depending on the compositional conditions of the natural gas produced. Zeolite membranes, on the other hand, are promising as CO_2_ separation membranes due to their excellent properties such as molecular sieving effect, selective adsorption, and high mechanical strength derived from their regular pore structure. Recently, zeolite membranes with an 8-membered ring pore size (3.8 × 3.8 Å), such as chabazite (CHA) and Deca-dodecasil 3R (DDR), have been successfully produced and have been reported to show high separation performance with separation factors exceeding 100 for CO_2_/CH_4_ mixture separation [4,5]. This is due to the fact that the kinetic diameter of the CO_2_ molecule (3.3 Å) is smaller than the pore size and that of the CH_4_ molecule (3.8 Å) is equal to the pore size of CHA zeolite.

High-silica CHA-type zeolite membranes exhibit high gas permeability due to their three-dimensional pore structure and large pore volume [6,7,8]. However, it has been reported that the CO_2_ permeability of CHA-type zeolite membranes decreases in high-pressure CO_2_/CH_4_ mixture gas systems [9,10]. Therefore, improved gas permeability is required for the application of zeolite membranes in high-pressure CO_2_ separation, such as in natural gas purification.

Grain boundaries formed during the formation process of zeolite membranes have been considered a problem to be solved for practical application as a factor that reduces the molecular sieving properties. Zeolite membranes have a polycrystalline structure in which zeolite crystals are densely deposited on a porous support, as mechanical strength and densification become problems without support [11]. In recent years, membrane formation methods that can suppress the formation of grain boundaries and repair methods after formation have been proposed [12,13,14]. Recent studies have attempted to quantitatively assess the influence of grain boundaries in Silicalite-1 (MFI) membranes by combining fluorescence confocal optical microscopy (FCOM) and image processing techniques, and have also shown that cracks (crystal cracks) are the biggest factor in reducing the molecular sieving effect of zeolite membranes [15]. The most important factor that reduces the molecular sieving effect of zeolite films is cracking [16].

However, it is difficult to completely remove grain boundaries from zeolite membranes, and the inherent performance of zeolites cannot be exceeded. The mechanism of gas separation at grain boundaries is also not well understood. We have reported the investigation of gas diffusion at grain boundaries inside MFI using the equilibrium molecular dynamics (EMD) simulation [17,18], but the effect of grain boundaries in mixed gas systems by EMD is difficult to determine due to the effect of the chemical potential gradient not being considered directly. On the other hand, the non-equilibrium molecular dynamics (NEMD) simulation can directly simulate gas permeation in membranes. For the direct simulation of membrane permeation, the dual-control volume grand canonical MD (DCV-GCMD) and simple NEMD have been reported. [19,20] We have used NEMD simulations to study the permeation mechanism of zeolite membranes with grain boundaries. In the process, it was found that zeolite membranes with slit-like controlled grain boundaries exhibit a higher separation performance than zeolite-specific CO_2_ separation performance [21]. In particular, CHA membranes controlled to have grain boundaries parallel to the direction of gas permeation were found to exhibit high CO_2_ permeability.

In this study, a perfectly crystalline model and a membrane model with controlled grain boundaries were prepared for a high-silica CHA-type zeolite membrane, and the effect of controlled grain boundaries on the CO_2_ permeability was discussed by simulating CO_2_/CH_4_ mixture gas permeation using the NEMD method at different pressure conditions. The NEMD results show that the presence of CH_4_ with slow diffusion within the zeolite crystals decreases the CO_2_ permeability, suggesting that the CO_2_ aggregation effect at the grain boundaries can be used to improve the CO_2_ permeability of the CHA membrane.

## 2. Calculation Method

**Figure 1:**
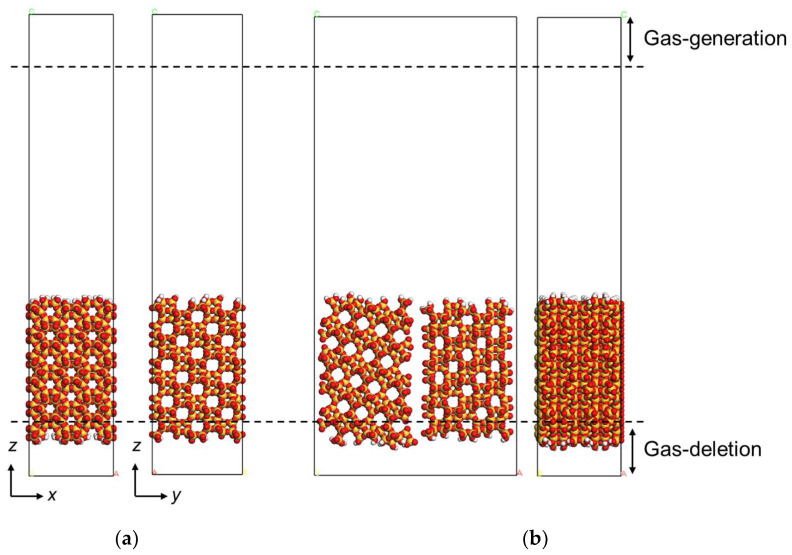
The cross view of CHA zeolite membrane on NEMD; (**a**) is a perfectly crystalline model and (**b**) is a polycrystalline model. The atoms by CPK model represent the silica atoms in yellow, oxygen atoms in red. The layer at the top and the layer at the bottom shows the gas-generation region and the gas-deletion region, respectively.

For the CO_2_/CH_4_ permeation simulation in CHA-type zeolite membranes, our previously reported NEMD calculation scheme was used, with atomic interaction parameters optimized to reproduce the experimental adsorption isotherm data [21]. In this study, the parameters of PCFF were applied for van der Waals between all atoms, and the atomic charges of silica, oxygen in crystal, and oxygen at the terminal were 1.244, −0.622, and −0.311, respectively. Figure 1 shows the unit cells of the perfectly crystalline model and the polycrystalline model in the NEMD. The gas molecules were generated on the generation region with a given velocity according to the Boltzmann distribution toward the membrane model, and permeated molecules were removed from the unit cell on the deletion region. This method makes it possible to control the number of gases impinging on the membrane on the feed and permeation sides.

The framework used a CHA-type zeolite structure with SiO_2_/Al_2_O_3_ = ∞, which contains no Al atoms at all, a perfectly crystalline model without defect, and a polycrystalline model consisting of surfaces with different crystal orientations fabricated. High-silica CHA membranes without Al are hydrophobic, which makes them very advantageous zeolite membranes for natural gas processing containing vapor [6]. The polycrystalline model was assumed to be a membrane with grain boundaries perpendicular to the surface. The grain boundaries in polycrystalline models were considered to be a slit pore with a width of 0.6 nm. The membrane thickness of all models was around 0.45 nm. The periodic boundary condition was used in two Cartesian coordinates along the membrane surface in the NEMD. To maintain the crystalline nature of the CHA zeolite, the coordinates of the zeolite’s constituent atoms, Si and O, were fixed during the NEMD. The oxygen atoms exposed to the surface were terminated with a hydrogen atom; on the other hand, the bared O atom on the grain boundary surface was not terminated with a hydrogen atom. Although the surface of the grain boundary of the CHA membrane would be terminated by the -OH group, because the test calculation of NEMD using the all OH terminated model showed no significant difference in the calculated membrane performance, the unterminated boundary surfaces were considered to be defined by the grain boundary size clearly.

The equimolar CO_2_/CH_4_ mixture was considered as the feed gas. Permeation simulations by NEMD were carried out by varying the time interval *f* for gas molecule generation, thereby varying the partial pressure conditions on the feed side (Equation (1)) [22].

(1) $$f=\frac{PS}{\sqrt{2\pi mk_{B}T}}\;$$

where *S* is the membrane surface area, *m* is the molecular weight of the gas molecule, $k_{B}$ is Boltzmann constant, and *T* is the temperature. All NEMD simulations were carried out at 298 K and the temperature of the system was controlled by the velocity scaling method. Table 1 summarizes the appearance intervals *f* used in this study. Gas molecules that were reflected at the membrane surface and far enough away from the membrane were deleted as not involved in permeation. The gas molecules permeating the membrane were removed just before they reached the permeate side and the pressure on the permeate side in all NEMD simulations was zero. The permeate flux *J* is calculated by counting the number of molecules, *n*, permeated during time Δ*t* at steady state and dividing by the surface area, *S*, of the membrane (Equation (2)).

(2) $$J=\frac{n}{\Delta t\cdot S}$$

## 3. Results and Discussion

### 3.1. Perfectly Crystalline Model

**Figure 2:**
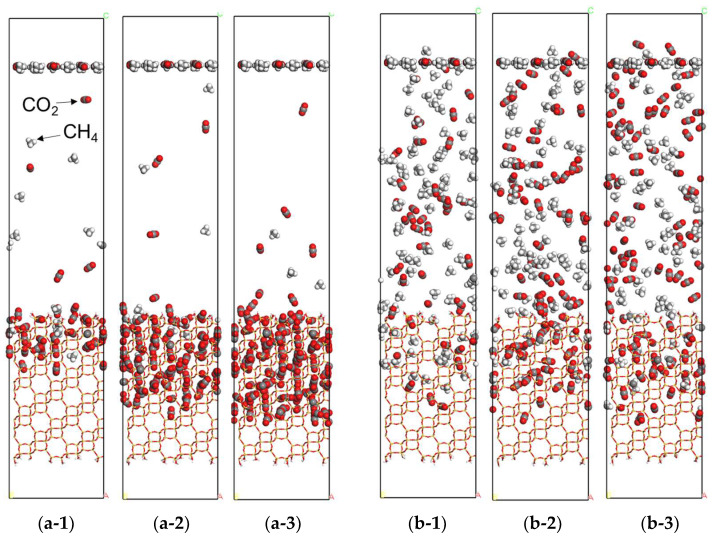
The snapshots of the unit cell at different simulated times were obtained from CO_2_/CH_4_ mixture gas permeation simulations using NEMD. CO_2_ and CH_4_ molecules by the CPK model represented the carbon atoms in gray, oxygen atoms in red, and hydrogen atoms in white, whereas the CHA zeolite was represented by the stick model. (**a**) 0.5 MPa, (**b**) 8.0 MPa, (**1**) 5 ns, (**2**) 10 ns, (**3**) 15 ns.

NEMD simulations of the CO_2_/CH_4_ mixture gas at different pressure conditions were performed on a perfectly crystalline membrane model of CHA zeolite with (1 0 0) crystalline planes oriented on the surface. Figure 2 shows snapshots of the NEMD results on the perfectly crystalline model at the feed pressures of 0.5 MPa and 8.0 MPa. Note that gas molecules are not shown in the snapshot as they are removed when they permeate the bottom of the zeolite membrane. Under the pressure condition of 0.5 MPa, CO_2_ molecules were observed to gradually permeate from the membrane surface, occupying the pores, irrespective of the orientation of the crystal planes. On the other hand, the number of CH_4_ molecules in the membrane was few. This is because of the molecular sieving effect of the membrane. The direction of the CO_2_ molecules in the membrane was parallel to the permeation direction and they were observed to be arranged in a row. This mode of diffusion is known as “single-file diffuse” [23]. This sequence is considered to indicate the direction of permeation of the CO_2_ molecules; the direction of the 8-membered ring pores in the CHA zeolite membrane. As the atomic interactions between the gas molecules and zeolite used in this paper were based on parameters that could reproduce experimentally reported adsorption isotherms [24], it can be assumed that CO_2_ molecules permeate according to the single-file diffusion within the zeolite crystals resulting in a high CO_2_ selectivity.

**Figure 3:**
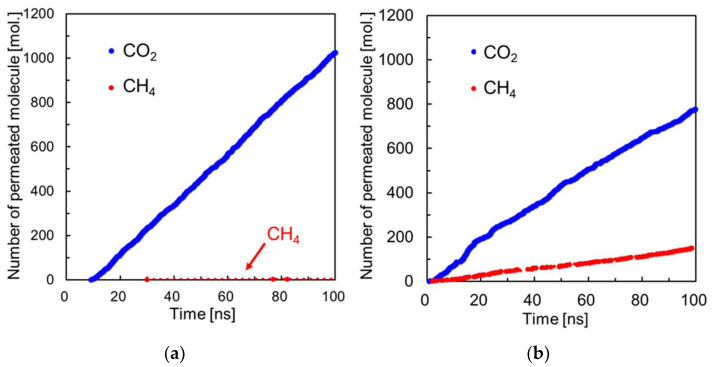
Changes in the number of permeated gas molecules through the perfectly crystalline model of CHA zeolite membrane. (**a**) 0.5 MPa, (**b**) 8.0 MPa.

As shown in Figure 2b, the NEMD at the feed side pressure of 8.0 MPa shows that the number of CO_2_ molecules in the membrane is lower than at 0.5 MPa. Figure 3 shows the change over time in the number of permeated gas molecules at 0.5 MPa and 8.0 MPa. The number of permeated CH_4_ molecules at 0.5 MPa was very small and it takes a longer calculation time (>100 ns) to calculate a precise flux. This may be due to the fact that in the defect-free, perfectly crystalline model, the kinetic molecular diameter of CH_4_ is almost equal to the pore diameter of the CHA zeolite, which requires a long calculation time for diffusion through the zeolite [25]. The number of CO_2_ molecules permeated increased almost monotonically after 20 ns, irrespective of the pressure conditions. At 8.0 MPa, the number of CH_4_ molecules permeated also increased monotonically after 20 ns. This is probably because of an increase in the frequency of CH_4_ impinging on the membrane surface at high pressure and an increase in the number of CH_4_ molecules entering the membrane pore. In other words, in a defect-free, perfectly crystalline CHA membrane, the permeation of CH_4_ molecules is significantly reduced at low pressure, indicating that although CO_2_, which is smaller than the pore size, is selectively permeated, this advantage may be lost at higher pressures.

**Figure 4:**
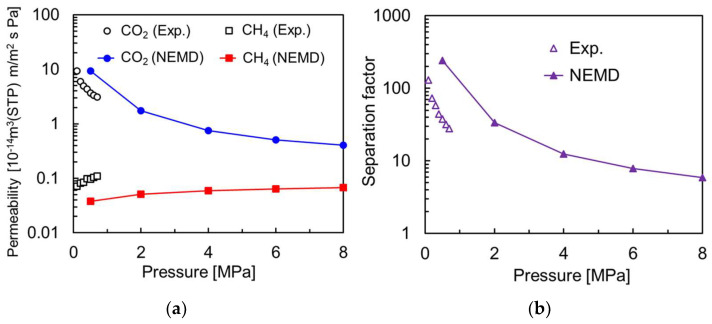
NEMD results on the perfectly crystalline model of zeolite membrane at different pressure conditions. (**a**) Permeability, (**b**) separation factor.

**Figure 5:**
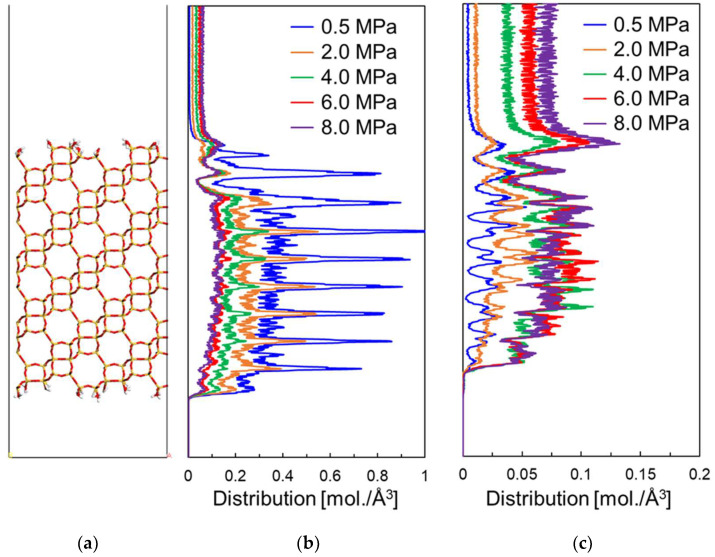
Concentration profile of gas molecules in the perfectly crystalline model on NEMD simulation at different pressure conditions. (**a**) Schematic of membrane model, (**b**) concentration profile of CO_2_, and (**c**) concentration profile of CH_4_.

Figure 4 shows the permeability and separation factor from the NEMD with the feed side pressures of 0.5, 2.0, 4.0, 6.0, and 8.0 MPa: the CO_2_ permeability decreases at higher pressures, while the CH_4_ permeability increases slightly. The results indicate that the CO_2_ selectivity decreases in the perfectly crystalline CHA membrane at high pressure. This is consistent with the trend in the experimental data of Kida et al. [9]. Analysis of the concentration distribution of gas molecules in the perfectly crystalline CHA membrane model for different pressure conditions on the feed side (Figure 5) shows that the CO_2_ concentration near the membrane surface on the feed side decreases with increasing pressure. On the other hand, the CH_4_ concentration increased at higher pressures, suggesting that the CH_4_ molecules adsorbed on the membrane surface inhibit the CO_2_ permeation.

**Figure 6:**
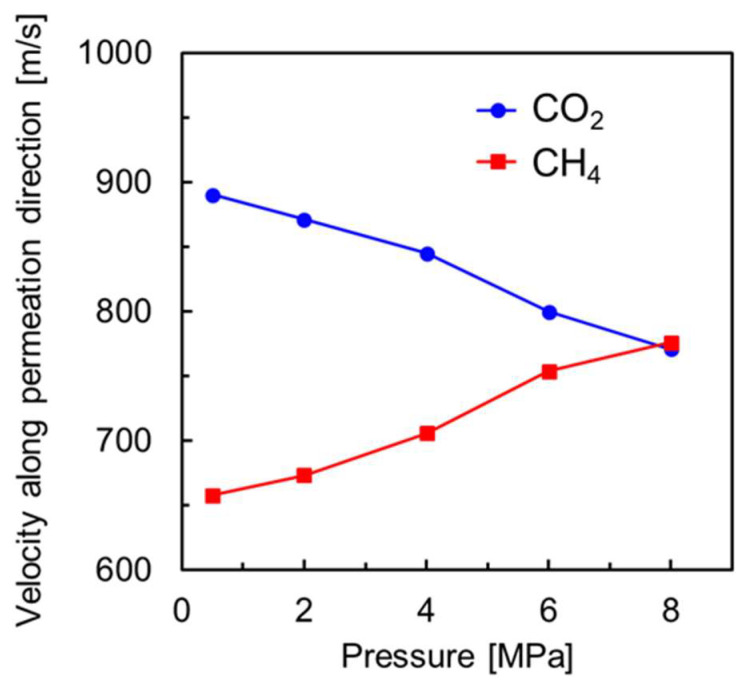
The velocity of gas molecules along permeation direction in a perfectly crystalline model of CHA zeolite membrane on NEMD simulation at different pressure conditions.

As shown in Figure 5, the molecular concentration of CH_4_ in the membrane tended to increase at high pressure and was almost the same above 4.0 MPa. On the other hand, the molecular concentration of CO_2_ in the membrane decreased at high pressure. This may be due to the fact that the permeation of CO_2_ molecules is inhibited by CH_4_ molecules, which diffuse more slowly in the pores, resulting in blocking the CO_2_ diffusion at higher pressures. Krishina et al. reported that the diffusion of gas molecules in zeolite membranes was strongly influenced by slower diffusing molecules [26]. Figure 6 shows the results of the analysis of the gas permeation velocity in the membrane with respect to the direction of permeation: at 0.5 MPa, CO_2_ molecules are permeated faster, while at higher pressures they are permeated more slowly. The higher the pressure, the slower the permeation velocity. On the other hand, the opposite trend was observed for CH_4_. At low pressure, the CH_4_ permeation is inhibited because CO_2_ molecules occupy the pores, but as the number of CH_4_ adsorbed in the membrane increases, the permeation of CH_4_ molecules, which diffuse more slowly, is considered to be the rate-limiting factor. A permeation test by Li et al. revealed that CO_2_ inhibits CH_4_ adsorption, and CH_4_ slows the CO_2_ diffusion in the SAPO-34 membrane that has pores that are similar to CHA [27]. In other words, it is clear that the contribution of diffusion within the zeolite crystals is greater than adsorption at the membrane surface to the reduction in the CO_2_ permeability in the CHA membrane at high pressure. Indeed, the adsorption selectivity of CHA-type zeolites in an equimolar CO_2_/CH_4_ gas mixture at 8.0 MPa is around eight [19,28], suggesting that the NEMD simulation results at 8.0 MPa show little CO_2_ selectivity due to diffusivity.

### 3.2. Polycrystalline Model

**Figure 7:**
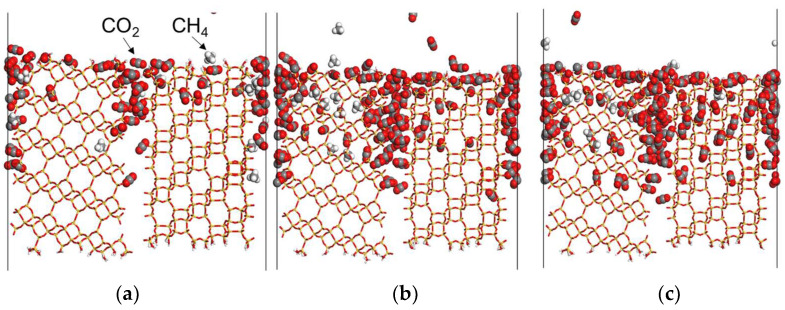
The snapshots of the cross view of the membrane in the unit cell were obtained from the NEMD for CO_2_/CH_4_ mixture gas permeation at high pressure. CO_2_ and CH_4_ molecules by the CPK model represented the carbon atoms in gray, oxygen atoms in red, and hydrogen atoms in white, whereas the CHA zeolite was represented by the stick model. (**a**) 5 ns, (**b**) 10 ns, and (**c**) 15 ns.

NEMD simulations of CO_2_ and CH_4_ mixtures were performed using a polycrystalline membrane model at 8.0 MPa. Similar to our previously reported results [21], selective aggregation of CO_2_ molecules was observed in the grain boundaries opened to the membrane surface. After 20 ns, when the number of permeated molecules becomes steady, CO_2_ molecules are selectively adsorbed at the grain boundaries rather than in the crystalline regions, as shown in Figure 7, and the permeability in the grain boundary membrane model is estimated to be a significant contribution of gas permeation.

**Figure 8:**
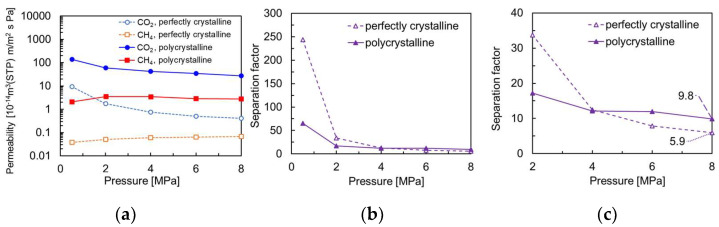
Comparison of NEMD results on the polycrystalline and perfectly crystalline membrane models at different pressure conditions. (**a**) Permeability, (**b**) separation factor, (**c**) enlarged (**b**) at high pressure.

**Figure 9:**
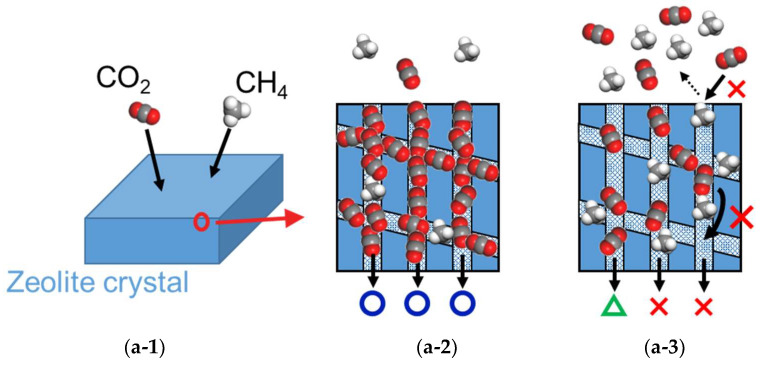

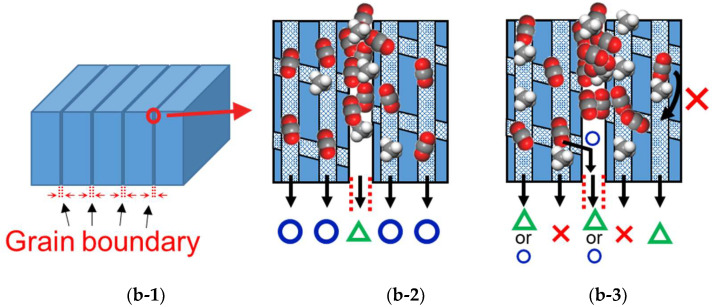
The illustration of the mechanism of effect in permeability by grain boundaries at low and high pressure. (**a**) Perfectly crystalline, (**b**) polycrystalline. (**1**) Schematic of membrane model, (**2**) low pressure, (**3**) high pressure, The symbols of the circle, the triangle, and the cross mean the high CO_2_ selectivity, low CO_2_ selectivity, and no selectivity, respectively.

Figure 8 shows the permeability and separation factors for the polycrystalline and perfectly crystalline membranes. The gas permeability in the polycrystalline model was more than ten times larger than in the perfectly crystalline membrane. The CO_2_ selectivity in the polycrystalline model at low pressure was smaller than in the perfectly crystalline model. This is due to the grain boundary being larger than the kinetic diameter of CH_4_, which reduced the molecular sieving effect, consistent with the trend obtained experimentally [8]. However, at pressures above 4.0 MPa in Figure 8c, the CO_2_ selectivity in the polycrystalline model was greater than that of the perfectly crystalline model. This is because the polycrystalline model has major CO_2_ permeation in the grain boundary region and is less susceptible to CO_2_ permeation inhibition by CH_4_, which occurs in the crystalline region. When separating CO_2_ from CH_4_ at high pressure using a high-density crystal membrane such as the perfectly crystalline model in this study, CO_2_ molecules have difficulty overtaking CH_4_ molecules in the CHA crystal. This is thought to be due to the one-dimensional diffusion of CO_2_ molecules between pores. On the other hand, the structure of the polycrystalline membrane modeled in this study is slit-like, which allows surface diffusion (two-dimensional diffusion) of CO_2_ molecules to overtake CH_4_ molecules. The illustration of mechanism of the effect by a grain boundary is shown in Figure 9. It is very difficult for CO_2_ molecules to overtake CH_4_ molecules on the permeation pathway in zeolite crystals because of kinetic diameters. On the other hand, in the presence of grain boundaries, there are permeation pathways from within the zeolite crystal to the grain boundary. This means that even if the CH_4_ molecules occupy the pathway within the crystal, the CO_2_ molecules can overtake the CH_4_ molecules by diffusing from the crystal to the grain boundary. Interestingly, the CO_2_ selectivity of the polycrystalline model at 8.0 MPa is greater than the adsorption selectivity (α = 8) in the CHA zeolite. At high-pressure conditions, the amount of gas adsorbed at the grain boundary is reported to be slightly greater than that of zeolite crystals [29]. Therefore, in CHA membranes with grain boundaries, CO_2_ molecules are not affected by the permeation-inhibiting effect of CH_4_ molecules within the crystals, and the polycrystalline membrane may have the ability to show higher separation performance than the perfectly crystalline model under high-pressure conditions. This means that fine-controlled grain boundaries may exceed the separation performance of zeolite crystals.

## 4. Conclusions

To investigate the CO_2_ separation performance of CHA zeolite membranes at high pressure, permeation simulations of a CO_2_/CH_4_ mixture gas up to 8.0 MPa were carried out using the NEMD. The NEMD results showed that CO_2_ permeation was blocked by the adsorption of CH_4_, which diffuses slowly inside the CHA zeolite membrane, and that this blocking effect reduced the CO_2_ selectivity at high pressure. On the other hand, in the CHA membrane with grain boundaries opened to the membrane surface, gases selectively permeated through the grain boundaries, thus suppressing the blocking effect caused by CH_4_ in the zeolite crystals. It was also suggested that the active utilization of fine-controlled grain boundaries can maintain CO_2_ selectivity and improve the permeation flux. This paper shows that grain boundary CHA films exhibit higher gas permeability than fully crystallized ones. These results demonstrate that precise control of the grain boundary structure can separate CO_2_ more efficiently than conventional zeolite membranes under high pressure.

## Figures and Tables

**Table 1:** Calculations of the time interval f for generating a gas molecule on each membrane model.

		*f* (fs)				
		0.5 MPa	2.0 MPa	4.0 MPa	6.0 MPa	8.0 MPa
Perfectly crystalline	CO_2_	21,524	5381	2690	1794	1345
	CH_4_	12,978	3245	1622	1082	811
Polycrystalline	CO_2_	9146	2287	1143	762	572
	CH_4_	5515	1379	689	460	345

## Data Availability

Not applicable.
